# Similar values, different expectations: How do patients and providers view ‘health’ and perceive the healthcare experience?

**DOI:** 10.1111/hex.13493

**Published:** 2022-04-12

**Authors:** Nabil Natafgi, Olayinka Ladeji, Shanikque Blackwell, Yoon Duk Hong, Gail Graham, Marcia Cort, C. Daniel Mullins

**Affiliations:** ^1^ Department of Health Services Policy and Management, Arnold School of Public Health University of South Carolina Columbia South Carolina USA; ^2^ Department of Pharmaceutical Health Services Research, School of Pharmacy, The PATIENTS Program University of Maryland Baltimore Maryland USA; ^3^ Mt. Lebanon Baptist Church HIV/AIDS Outreach Services Baltimore Maryland USA; ^4^ University of Maryland Capital Region Health Lake Arbor Maryland USA

**Keywords:** health values, patient engagement, patient experiences, patient–provider expectations, urban communities

## Abstract

**Introduction:**

No one can argue on the importance of health in one's life. However, the value of health in the context of other priorities for individuals is not always as clear. Further, patients' experience with the healthcare system is rarely contrasted with the service providers' expectations. The aim of this paper is to examine and compare patients' and providers' own definitions of health and their perceptions of the healthcare delivery experience from the lens of residents and providers in West Baltimore, Maryland.

**Methods:**

This was a qualitative study with semi‐structured focus groups (15 sessions) and individual in‐depth interviews (21 interviews) with 94 participants. Two independent coders thematically analysed the transcripts.

**Results:**

Patients identified five areas where health systems can help them stay healthy or become healthier: affordability and costs of care; accessibility; clinician/patient communication; addressing social determinants; and stigma and trust. Providers acknowledged that the healthcare experience is not always perfect. While the medical team focuses on conversations that enhance medical care, patients are expecting providers to touch on subjects beyond medical care.

**Conclusions:**

Patients and providers need to consider that although they have a common value towards health, there is still a gap in what users expect and what providers can offer. To further align those expectations, there is a need for increasing involvement of patient in care administration and improving dialogue between the parties about these differences.

**Patient or Public Contribution:**

A Stakeholder Advisory Board (SAB)—comprised of a patient, two community leaders, a physician and two healthcare administrators—was instrumental in codeveloping the study material (e.g., interview guides), engaging patients in the research process, identifying participants and codeveloping dissemination material. Two SAB members—Gail Graham, a patient consultant/professor, and Marcia Cort, a physician—are coauthors.

## 1 INTRODUCTION

For decades, the World Health Organization has defined health as the ‘state of complete physical, mental and social wellbeing and not simply the absence of disease or infirmity’.^1^ By defining health so broadly, there are three traits that are often considered when talking about health: (1) the lack of (physiological or psychological) illness, (2) the ability to handle the demands of daily life and (3) the balance between one's self, social dynamics and the physical environment.^2^ Out of the three ways of describing health, the third descriptor is probably the most important as it emphasizes the factors that affect an individual's mental health, social standing, access to resources (including healthcare services) and ability to achieve the highest quality of life. Understanding these interrelated factors is instrumental in helping patients, physicians and policymakers create a health system that provides quality care.

As the importance and impact of quality of care have grown, many studies investigated how the intersection of the beliefs and values of patients and the perception of providers impact the healthcare system and the type of care provided.^3^, ^4^, ^5^ This line of research has shown that there is often discordance between patients' health beliefs and values and providers' perception of the patients' health beliefs and values.^5^ Provider and patient perceptions often lack concordance in terms of perceived physical and psychosocial health status and needs as well as the use of resources and support available for disease self‐management.^6^, ^7^, ^8^, ^9^, ^10^ Research has also revealed that healthcare providers often diverge on perceptions of emotions, satisfaction and opinion of the quality of communication during a medical visit, quality of chronic illness care received by patients and perception of stressors while receiving care in the healthcare setting.^11^, ^12^, ^13^

When patients' beliefs and values do not align with the providers' perceptions, patient experiences often suffer. These negative patient experiences are especially evident in the encounters of African American patients with the healthcare system. The African American patient experience is oftentimes underscored by feelings of mistrust towards medical providers.^14^, ^15^ Two notable occasions that have resulted in mistrust in the healthcare system are the Tuskegee Syphilis study and Henrietta Lacks.^16^, ^17^ Both of these instances demonstrate historical moments in which health systems discriminated against and took advantage of African American patients without their consent.^18^ This feeling of unease remains a reality for many minority patients today, including African Americans. For instance, a recent systematic review of minority patient preferences, barriers and facilitators for shared decision‐making with healthcare providers showed that African Americans were not being prepared for a discussion with their providers and were less comfortable in taking on an active role in their own health‐related decisions.^19^

### 1.1 Conceptual framework: Learning health systems

The study, overall, and the interview guide were primarily based on the Learning Health System (LHS) Framework and its reflection in communities, through the novel Learning Health Care Community (LHCC) model. The LHS model framework has three foundational elements: health‐related data generation, performance improvement targets and a supportive environment.^20^ The LHS model works by ‘systematically capturing and translating information generated by clinical research and health care delivery’ to close open‐ended learning loops.^21^ The LHCC model builds on the LHS framework by bringing the LHS's core components into the community by continuously engaging residents, patients, providers and other community stakeholders in the learning process.^22^, ^23^

### 1.2 Study objectives

As evidence has mounted to compare and contrast the perceptions of patients and providers, very little research has been done to contrast the experiences of patients within the healthcare system with the expectations of providers, particularly from the perspectives of those who are part of a minoritized population. The overall aim of our study was to gain community members' insight regarding how to make the LHS concept more community‐focused and identify strategies that can address the needs of service users' and understand the gaps that exist between their experience and the perceptions of their providers. The objective of this paper, specifically, is to contribute to the health expectation literature and add to the knowledge regarding service users' experience with the healthcare system as compared to the perception of providers from the lens of residents and providers in West Baltimore, Maryland.

## 2 MATERIALS AND METHODS

### 2.1 Stakeholder Advisory Board

This study was designed and informed by community‐based participatory research approaches with the goal of gathering and studying qualitative data to understand the differences in the perception of patients and healthcare professionals when it comes to patient–provider interactions. The first step in this process involved the assembly of a diverse, inclusive and representative Stakeholder Advisory Board (SAB). The SAB members were purposively chosen to reflect a diverse set of experiences and knowledge that can inform the study goals and board function.^24^ With this in mind, we identified individuals and entity representatives who can serve on the board and provide appropriate guidance. To facilitate group meetings, ensure efficiency and stay on budget, we decided to invite six members to join the SAB, with equal representation of patient/community advisors including three members and professional advisors (three members). All invited members were offered compensation for their time and agreed to serve on the SAB.

The SAB included (1) Jacqueline (Jackie) Caldwell, President of Greater Mondawmin Coordinating Council (GMCC, an umbrella council of neighbourhood associations in West Baltimore) and Baltimore Civic Site Administrator at Annie E. Casey Foundation (*Community Leader and representative of West Baltimore residents*), (2) Damion J. Cooper, M.Th, Founder/Executive Director of Project Pneuma and Director of the Office of Neighborhood Relations at Baltimore City Council (*Community Leader and representative of Baltimore residents*), (3) Gail Graham, Director of Mt. Lebanon Baptist HIV/AIDS Outreach Services and Global Representative at Johns Hopkins Community Advisory Board for HIV/AIDS Clinical Trials (*Patient Advisor with living experience in challenges in accessing healthcare services*), (4) Jennifer Baldwin, RN, MPA, Senior Vice President for Patient‐Centered Medical Homes at CareFirst BlueCross BlueShield (*Payers' Representative*), (5) Marcia Cort, MD, MBA, FACEP, FAAEM, Chief Medical Officer at Total Health Care, Inc (Federally Qualified Health Center in West Baltimore) (*Providers' Representative*), and Karen Kippen, MSA, Executive Director of Patient Centered Outcomes Research at Henry Ford Health System (*LHS and Researchers' Representative*).

The purpose of the SAB was to collaborate and lend their collective voice across the continuum of this project. The SAB members were instrumental in (a) codeveloping study material (e.g., interview guides), (b) brainstorming how to actively engage patients in the research process, (c) offering guidance on implementation and timing to ensure that the research process makes sense to patients, (d) assuring that the language used is culturally appropriate, (e) helping identify and recruit participants representative of the target population, (f) codeveloping scientific and lay publications to disseminate findings and (g) advising on media forms and outlets for dissemination.

### 2.2 Data collection

Focus group sessions and in‐depth interviews were conducted to hear directly from patients, community members, healthcare professionals and administrators and other stakeholders regarding their views on the concept of health and healthcare experiences.

#### 2.2.1 Interview guides

Following an iterative process, the investigators, along with the SAB, codeveloped a semi‐structured interview guide (see Documents S1 and S2) to identify themes and questions that would increase understanding of patients' and healthcare professionals' viewpoints on health and healthcare, their experiences with the healthcare system and patient–provider interaction. A scoping search of the literature (focused on the concepts of LHS and LHCC) identified a roster of themes and questions that can potentially be included in the interview guide. This roster of themes and questions was then shared with the SAB members, who collectively identified the top concepts to be addressed and the questions to be asked under each section. Based on the recommendation of the SAB—particularly the patient and community representatives—the term ‘doctor(s)’ was used to refer to healthcare professionals in the interview guide. The rationale for the use of this terminology is that the many community members think of ‘doctors’ as the providers of care (even if, for instance, they were physician assistants or nurses, etc.) and that they would be more familiar (and responsive) with the term ‘doctor’ than the term ‘healthcare provider’ or ‘healthcare professional’. Two separate versions of the guide were developed with some slight modification in the question framing: one addressing patients and community members and the other addressing healthcare professionals. The interview guide was pilot‐tested with a group of 15 participants, one focus group and five interviews, to refine questions to meet our needs.

#### 2.2.2 Recruitment and participants

The investigators used purposive sampling to identify and recruit participants who could provide comprehensive insight into our research questions, with support from SAB members, various key partners and grassroots on‐ground outreach efforts (e.g., snow‐ball recruitment techniques, flyers distributed in community resource centres, at health fairs and other community outreach events). Participants' demographics were collected using a separate survey administered before the interview or focus group sessions to assess and ensure diversity of participants in terms of age, gender, race, educational attainment, self‐rated health and health coverage. Recruitment of participants continued until we reached saturation in themes within respective stakeholder groups. A total of 94 participants participated in 16 in‐depth interviews and 15 focus groups between June and September 2018.

#### 2.2.3 Focus group and interview process

The focus group sessions and in‐depth interviews were moderated by researchers trained in qualitative methodology and were conducted in private spaces and lasted for approximately 60–90 min. Each session began with the participants receiving information on the project and being informed that their participation was voluntary and that they were free to leave or decline to answer a question at any time. With their consent, each of the discussions was audio recorded to supplement the field notes. The audio‐recorded interviews were transcribed by a professional transcription service. We also compensated each participant with a $25 gift card for their time.

### 2.3 Coding process and analysis

A two‐step coding process was followed to analyse the data in concordance with similar qualitative approaches.^23^, ^25^, ^26^ The first step was inductive, where two coders independently read each transcript to identify emerging themes, followed by a group meeting to discuss extracted themes, identify broad categories and refine them into headings that matched the original concepts outlined in the interview guides.^27^, ^28^ Under each category, major themes and sub‐themes were identified and presented in a codebook that was used for the second step. The second step was deductive, where an inductively developed template/scheme was applied to re‐code all the data in a uniform fashion and extract quotes related to each of the themes identified in the first step. Each quote was then reviewed independently by a different coder to confirm thematic relevancy. Differences were reconciled in a group meeting until a consensus was reached.

The reliability of the data collection and analysis was assessed using Lincoln and Guba's^29^ criteria. Credibility was verified by a member‐checking procedure, where identified themes were shared with a random sample of participants (15%) to assess whether our data interpretation and emerging themes are an accurate representation of their experiences.^30^ Transferability was achieved by sharing the methodology, quotes and detailed descriptions of the context in all disseminated material. For dependability, we asked the SAB members to examine both the process and the product of the research study and evaluate the findings, interpretations and conclusions. Lastly, confirmability was realized by analyst triangulation, with multiple analysts being involved in the data collection, coding, analysis and review of findings.

A summary of the results was shared with participants who opted to receive study findings/summary at the conclusion of the study. The study was approved by the Institutional Review Board at the University of Maryland, Baltimore, and followed the consolidated criteria for reporting qualitative research (COREQ) guidelines.^31^

## 3 RESULTS

A total of 94 participants participated in 16 in‐depth interviews and 15 focus groups. *Thirty‐nine* participants (41%) self‐identified their primary role on the interview as community member/resident, community leader or employer, *twenty‐four* (26%) self‐identified as patient, patient advocate or caregivers and *thirty* (32%) self‐identified as healthcare professionals, healthcare administrators, healthcare payers/insurers, health services researchers or health profession students. One participant chose not to disclose their primary stakeholder role. For the purposes of the demographics and qualitative analysis, the first two stakeholder roles (community members/leaders and patients/caregivers) were collapsed together and labelled as ‘service users’. It is noteworthy that when discussing patient experiences, we believe that it is important to use terminology that allows us to be inclusive of community members and leaders, patients and patients advocates who utilize healthcare services. To this end, and for the purposes of this paper, we use ‘patients’ and ‘service users’ interchangeably, with the understanding that community members and leaders also use healthcare services and interact with healthcare professionals.

Slightly less than half of the participants (45%) were between the ages of 45 and 64 years, and the majority (82%) self‐identified as Black or African American. The majority of healthcare professionals (70%) had more than 4‐year college degree, while the service users had a normal distribution of educational attainment. Further, almost two‐thirds of the healthcare professionals (63%) rated their health as very good or excellent, while only two‐fifth of the service users (40%) did so (Table 1).

**Table 1:** Focus group and in‐depth interview participants' characteristics^a^

Participants' characteristics	Service users (*N* = 63)	Healthcare professionals (*N* = 30)
Age group		
18–44 years	12 (19%)	14 (46.7%)
45–64 years	28 (44.4%)	12 (40%)
≥65 years	21 (33.3%)	3 (10%)
Undisclosed	2 (3.2%)	1 (3.3%)
Sex		
Male	17 (27%)	7 (23.3%)
Female	43 (68.3%)	23 (76.7%)
Transgender	3 (4.8%)	
Race		
Black or African American	57 (90.5%)	18 (60%)
White	3 (4.8%)	6 (20%)
Other	1 (1.6%)	5 (16.7%)
Undisclosed	2 (3.2%)	1 (3.3%)
Education		
Some high school or less	6 (9.5%)	1 (3.3%)
High school graduate or GED	14 (22.2%)	5 (16.7%)
Some college or 2‐year degree	17 (27%)	3 (10%)
4‐year college graduate	11 (17.5%)	21 (70%)
More than 4‐year college degree	13 (20.6%)	
Undisclosed	2 (3.2%)	
Self‐rated health		
Poor or fair	12 (19.1%)	1 (3.3%)
Good	24 (38.1%)	8 (26.7%)
Very good or excellent	25 (39.6%)	19 (63.3%)
Undisclosed	2 (3.2%)	2 (6.7%)
Health coverage		
Yes	60 (95.2%)	28 (93.3%)
No		1 (3.3%)
Undisclosed	3 (4.8%)	1 (3.3%)

^a^ The table does not include the demographic information of the 15 individuals who participated in the pilot interviews and focus groups. These individuals were excluded because the demographic questionnaire was not created at the time of the pilot. We also excluded the demographic information for one participant because they chose to not disclose their stakeholder role.

The themes and findings from the interviews and focus groups can be classified into two main categories: Concept of Health and Healthcare Experiences. Service users' and service providers' own definitions of health and their perceptions of the healthcare delivery experiences demonstrate differences in what users expect and what providers can offer. The following section summarizes the various emergent themes and discussions relevant to each of those two categories (concept of health and healthcare experiences), stratified by service users vis a vis service providers.

### 3.1 Concept of health

‘Do you want to live?’ This was one of the questions that we posed to participants at the request of the SAB members. This question was selected, not for the direct answer, but to elicit critical thinking and more elaborate responses. Almost all respondents expressed an absolute desire to live. Mostly, participants wanted to live a healthy and productive life for themselves and their families. In addition, more than one participant emphasized that they also focus on the quality of life and not only years lived—‘not just existing, want to go beyond existing’.

#### 3.1.1 Health as a priority

When asked, ‘among the things that are of value to you, where do you place your health? Your family's health?’ many participants stated that it was at the top of their list. Participants specified that health is a top priority because one cannot take care of their family if they are not healthy. Several other responses were that health was a top priority because being healthy is the key to living life and if you are not healthy you cannot do the things that you want to do.

When addressing health as a priority, one needs to understand the distinction between younger and older populations. For instance, younger participants (e.g., students) did not place health as high on their list because they felt they are young and healthy so they do not have to actively think of it and prioritize it. Among students, for example, their education may be their priority at that stage of their life and career. Another important distinction is between theory and practice. A couple of participants (namely healthcare professionals) clarified that as healthcare professionals, they realize the importance of health and that it needs to be their priority in life. However, in practice, they believe that other life necessities come into play (e.g., work–life balance, finances, enjoying life, spirituality or religion, etc.).

Different participants had different motivations to stay healthy or become healthier. The top two motivators were ‘living longer’—driven by the need to feel well and prevent future long‐term illness—and ‘family’—driven by the urge to stay around for family members and see their kids and grandkids grow up. Participants stated that they were motivated by their desire to be present for their family and wanting to set a good example for those around them. One participant stated that, ‘sometimes, I want to give up… I want to do it, but then you think, “I have kids, grandkids. They need me and love me, and I got to keep fighting”. So, I just keep fighting…’ Some also voiced a fear of not being healthy due to the experiences that they have seen of loved ones, community members, on advertisements and in hospitals/clinics. Other motivators included spirituality (e.g., God or religion), witnessing examples (e.g., healthy role models to follow, or sick individuals to avoid following), self‐worth (e.g., self‐motivation, self‐love, self‐care) and upbringing (e.g., raised to seek good health).

When asked to identify how they reward themselves for selecting healthy choices, participants identified two main subthemes: first, *self‐reward*, by doing enjoyable activities (e.g., getting hair done) or treating oneself (e.g., burgers, a romantic dinner, cookies, smoothies, cheat day), and second, *self‐encouragement* or *self‐satisfaction*, by being rewarded through the good outcomes that they witness (e.g., lower weight, lower blood glucose level). These rewards were oftentimes a pat on the back or the manifestation of good health outcomes.

Participants acknowledged that not all individuals would necessarily value healthy lives the same way. Four themes emerged to the question ‘How can we encourage people to focus on their health when they are healthy (i.e., not sick)?*’* Recurring themes were related to education, spreading awareness and providing opportunities to engage in a healthy lifestyle. Participants felt that it was necessary to let people know that health is important and that they ‘should take care of themselves from a state of wellness and not from a state of sickness’. Other themes emerging in relation to this question included communication (e.g., story sharing and talking openly about health), overcoming barriers to information or to care (e.g., trust in healthcare system, affordability and access, hard‐to‐reach populations, connection with health and living), and setting examples (both good and bad). One participant explained that dissatisfaction with healthcare providers could prevent patients from getting the care that they need to live, relaying a story of a young man who refused medical treatment for a gunshot wound because of a lack of trust in the healthcare system and instead got gauze from a community pharmacy to self‐treat.

#### 3.1.2 Health needs

In general, participants seemed to be aware of their health needs, but the source of this knowledge seemed to vary. Some indicated that they learn from their care providers (e.g., through primary care visits), while others learn about their health through their family and friends, or other individuals with similar health conditions (e.g., asking what medications have you taken? what feelings did you have from that? did you experience a difference? are you trying something on your own?). Some learn through media (e.g., TV, magazines, bus advertisements, and social media) or through self‐learning and/or self‐awareness (e.g., internet surfing, monitoring blood glucose level, weight measurement). Other sources of learning about health and health needs, as mentioned by participants, included health fairs, faith‐based organizations (e.g., churches) and educators who make the information easy to understand. On this note, few participants mentioned the information overload that they receive sometimes, exemplified through often‐conflicting information (e.g., opposing study results) or inability to discern the reliability of information.

**Figure 1:**
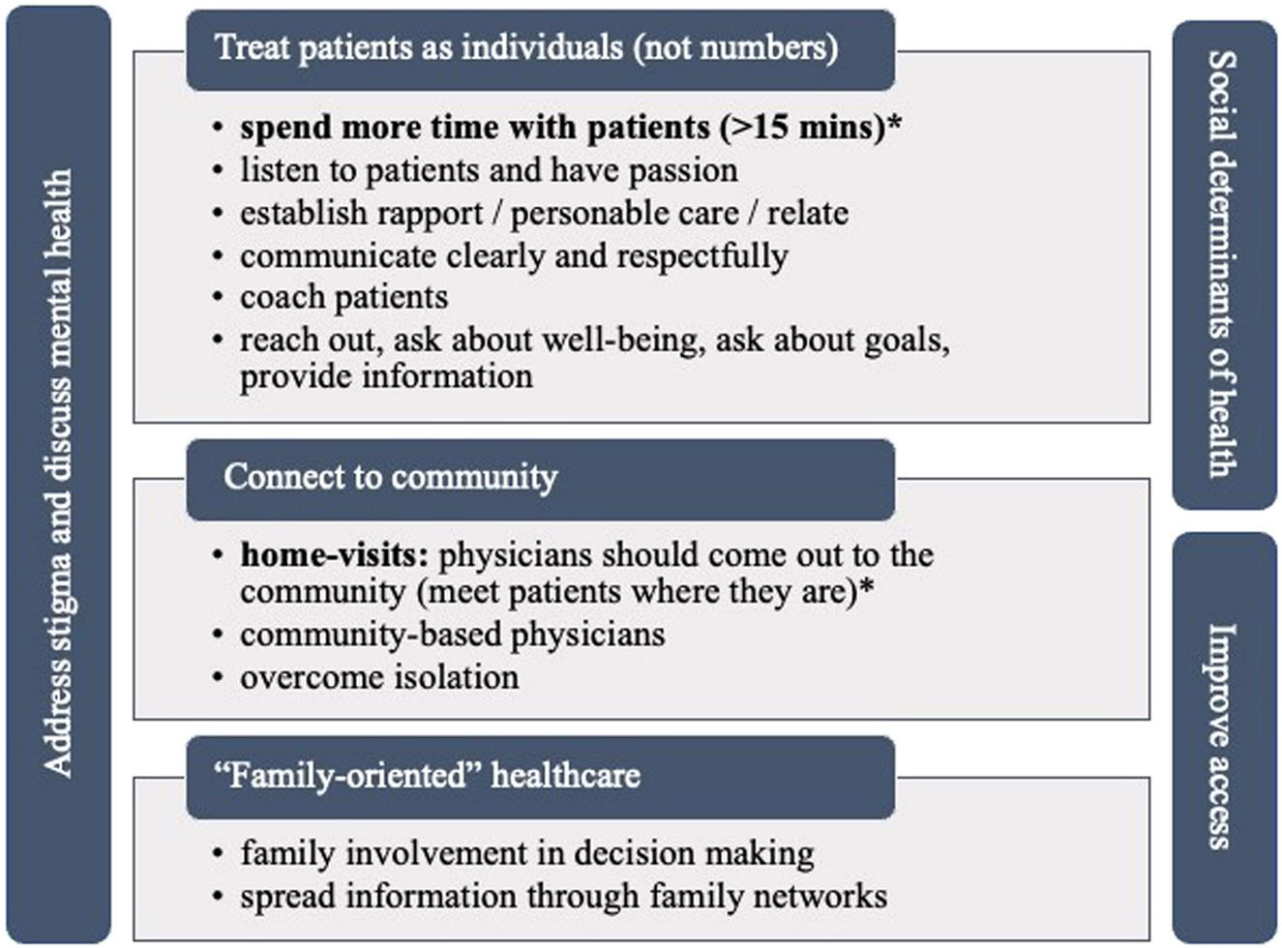
Concepts of health—what can providers and health systems do differently? *A commonly recurring theme

When asked ‘What can doctors and hospitals do differently to help you become as healthy as possible? How can they support you to stay healthy?’ responses focused on six dimensions: (a) enhance communication and treat patients as persons/individuals (not numbers), (b) connect to the community, (c) provide ‘family‐oriented’ healthcare, (d) improve access and affordability, (e) address social determinants of health and (f) overcome stigma (Figure 1).

*Treat patients as persons*. Participants expressed a desire for doctors to demonstrate that they care. One patient noted that ‘Over the past 15–20 years, we [have] gone into “fast‐food” type of medical care’. Patients indicated that providers should develop a relationship with their patients and aim for more personalized care visits. They would like to see healthcare providers spending more time with the patients, listening to their needs, relating with their realities and establishing rapport. In reality, though, providers also acknowledge this need, as a healthcare professional stated: ‘In medicine, we get this whole mindset of 15 min per patient, so unfortunately doctors don't get to ask those important questions that aren't necessarily related to the reason they are coming in, but are very important to their overall health’.

*Connect to community*. Another consistent theme is related to home visits. Quite a few participants, both patients and healthcare professionals, talked about physicians going back to seeing patients in their home: ‘I think it helps for the physician to come out of the building and into the community. Because then you can also see the environment the person is in. That really helps. Because if you're giving someone advice while they're in your office that's all well and good but if you go to their house and see the reality, how can they take insulin if they don't have gas and electric on? You can't put anything in the fridge!’ In parallel, providers also shared a similar call to overcome isolation and move towards more community‐based care and home visits. They emphasized the importance of providers reaching out into the community and finding people where they are, instead of making patients come to them.

*Provide ‘family‐oriented’ healthcare*. ‘Family‐oriented’ healthcare was key for patients looking for more involvement (as patients themselves and their family members/friends) in the decision‐making process. Participants also reiterated the significance of spreading information through family networks: ‘I think a particular emphasis on family is an important concept. Some of the most successful interventions have occurred because families have been trained to know how to do a proper intervention’.

*Improve access and affordability (cost) of care*. A couple of participants expressed frustration over shutting down of hospitals in their areas without replacement and called for facilities that are affordable and accessible so that they do not have to ride out to central larger medical centres. They also requested that physicians have more appointments available. Nonetheless, it was also noted that that not all patients keep their appointments and that there are a lot of no shows, which calls for a change in patients' mindset of what they think of doctor visits—‘think of it as an interview’. Participants also pointed to the prohibitive nature of out‐of‐pocket payment to receive healthcare, noting that if healthy people are to be regularly screened and tested, then such preventive services should be offered free (or at least be affordable).

*Address social determinants of care*. Participants clearly established a link between social determinants and their health outcomes. In particular, they noted two factors that affect one's access to healthy living: housing (or lack thereof) and drug programmes in the community. Still, they acknowledged that this is a collective responsibility, but healthcare systems should be a part of addressing social determinants of care as well.

*Overcome stigma*. Some participants also pointed out to the stigma associated with some diseases. They clarified that some individuals might not be comfortable sharing information about their disease or symptoms, even with healthcare providers. For example, men might be uncomfortable undergoing a colonoscopy for colon cancer screening. They suggested that providers should establish rapport with patients, so that they feel comfortable sharing information that is sensitive or for undergoing uncomfortable tests. Moreover, participants emphasized the role of health systems and providers in addressing mental health issues in the visit as well. Health systems, participants said, should have a team (support group) or videos (learning modalities) to help patients with their disease and address their mental health condition.

### 3.2 Healthcare experiences

#### 3.2.1 Service users' experiences

When asked to think back to a great experience that they had with healthcare experiences, most shared occurrences where they felt that they received personable care, and care that made them feel empowered and listened to. These experiences were explained as a time when the provider took their time and explained their health conditions. The providers would also ask them how they were doing and listened to the patients' knowledge about their condition. One participant described the experience as ‘when he goes in to see a patient that [white] coat ain't [sic] on. It's just, “How [are] you doing today, I'm Dr. So‐and‐so. I'm here to help you out. How [are] you feeling today? What's going on with you?” He comes at them like that, and I respect him because he took off his coast. It makes them feel more comfortable’. These positive experiences were also categorized by the physicians following up with patients regarding their care and being available when patients reach out for additional information. Another theme that was present in participant responses was relationships. When describing their great experiences with health systems, patients felt that relationships that demonstrated that the physician was invested, respected them and allowed them to open up about the situations in their life were the most memorable and impactful. According to participants, building this rapport encouraged them to be more vocal about the health issues that they were facing.

Even though a few participants shared great experiences that they had with the healthcare system, most of the service users recalled bad experiences. In the unpleasant experiences that were shared, service users expressed feeling rushed by the physician and not being seen as an individual. When describing these instances, service users stated that doctors did not take the time necessary to build a relationship with the patient and they did not ask ‘How are you?’ The majority vocalized that this demonstrated a lack of compassion. It was also stated that oftentimes, providers underestimated the patients' ability and knowledge about their health conditions/experiences. In discussing these occurrences, some patients stated ‘I don't like arrogant [providers] who walk into a situation and assume they know what's best for me. [Providers] can't always know what's best for me without at least having a conversation’. These bad experiences can also be categorized on the basis of the lack of staff available at the doctor's office (Table 2).

**Table 2:** Themes emerging in response to questions about health experiences

**Great experiences**	
Personable care	‘Her pediatrician actually called us every 2‐3 hours to see if she was eating, to check on her. That spoke a lot to his character. He was a very busy man, he didn't have to keep calling us. […] That made us think about medicine as more than just a name on someone's lab coat’. Parent of a paediatric patient
Authenticity/genuine care	
Friendly/reassuring/comforting	
‘Knowing’ the patient/rapport	
Quality of care	‘I liked how they got things done on time and kept up on it’. Patient ‘Some people don't understand certain medical terminology, you have to make their clients comfortable enough’.
Accurate/early diagnosis	
Quality of communication	
Timeliness of care	
Trust in care provided	‘I've been fortunate that, in general, when you have a great relationship with your patient that it's easier to be able to build up that rapport for them to be able to trust you, take information from you, bring information to you, and get to the outcomes that are beneficial […] In general, if you're up front and honest with people, they can see that’. Healthcare professional
Patient empowerment	
Shared decision‐making	‘And then on the other hand, if it's a patient that doesn't want the medication, they're taking charge of their health too, so they have to listen to them as well’. Healthcare professional
	‘I felt in control because […] they shared information with me. They didn't dictate, they just made sure I understood’. Service user
**Negative experiences**	
Not listening/assume patient lacks knowledge	‘I had gone to another doctor to discuss the same problem and he barely listened to me. I tried to explain the history and he kept assuming what could be wrong’. Service user
Lack of compassion	‘Not going in as though, “I'm here to do this job and go”. I need you to relate to me. So that's my biggest fear. How are these professionals going to go into the community?’ Service user
Lack of rapport	‘In medicine, we get this whole mindset of 15 minutes per patient, so unfortunately doctors don't get to ask those important questions that aren't necessarily related to the reason they are coming in, but are very important to their overall health’. Healthcare professional
	‘One way is to stop being so patronizing and condescending to patients. Talk to them as if you know them’. Service user

When asked what providers and healthcare systems can do to improve the healthcare experience, service users stated that doctors could ask how the patient is doing. In addition to this, they stated that doctors could also ask them about the social determinants of health. This includes questions about their mental health and trauma, their family, financial burdens and social support. One patient emphasized the importance of such dialogue by stating, ‘If you have a bunch of homeless people, health isn't at the top of your list. Eating and having shelter would be at the top of the list’.In addition to the social determinants of health, service users also voiced a desire for providers to meet them where they are and ensure that they understand the information that was discussed during the visit (Table 3).

**Table 3:** Themes from service users and healthcare professionals in response to question about improvements in care

**Service users**	
Establish rapport	How are you really feeling? Not just, ‘How are you feeling?’
Social determinants of health	‘I would have liked it if he had taken a moment to ask about my mental health’
	‘I might ask a patient about other social aspects that may prevent them from caring for their health the way they should’.
Understanding	‘Do you understand what I just told you? Do you understand what—like a teach‐back, kind of thing’.
**Healthcare professionals**	
Affordable	‘Sometimes people aren't compliant because they can't afford it. We look to help them with programs that can offer patient assistance or educating them on how to use it appropriately so that it's easier for them’.
Access	‘What their barriers are to accessing health care’.

#### 3.2.2 Service providers' experiences

On asking service providers to describe their great experience with a patient, most of the interactions described were ones in which they had built relationships with their patients. These scenarios were characterized by the providers feeling like they had helped the patient learn about their condition. They felt like they had empowered the patient to take control and ownership of their health condition and treatment regimen. They increased their knowledge of the medication they were taking and the symptoms. This is demonstrated by one service provider saying, ‘One of the most rewarding was maybe… they might have been on a certain medication or something for years and then they come in, we provide education and they realize they have been using it incorrectly for years’. Taking those steps to connect with the patient led to physicians feeling as if they had seen a shift in the patient's mindset. Another observation is that service providers did not reference any bad experiences they may have had with their patients. When asked about the ways in which healthcare can be improved, service providers listed out ideas related to the system of care. They reported that they would improve access to care and make it affordable for patients.

#### 3.2.3 Questions doctors did not ask

**Figure 2:**
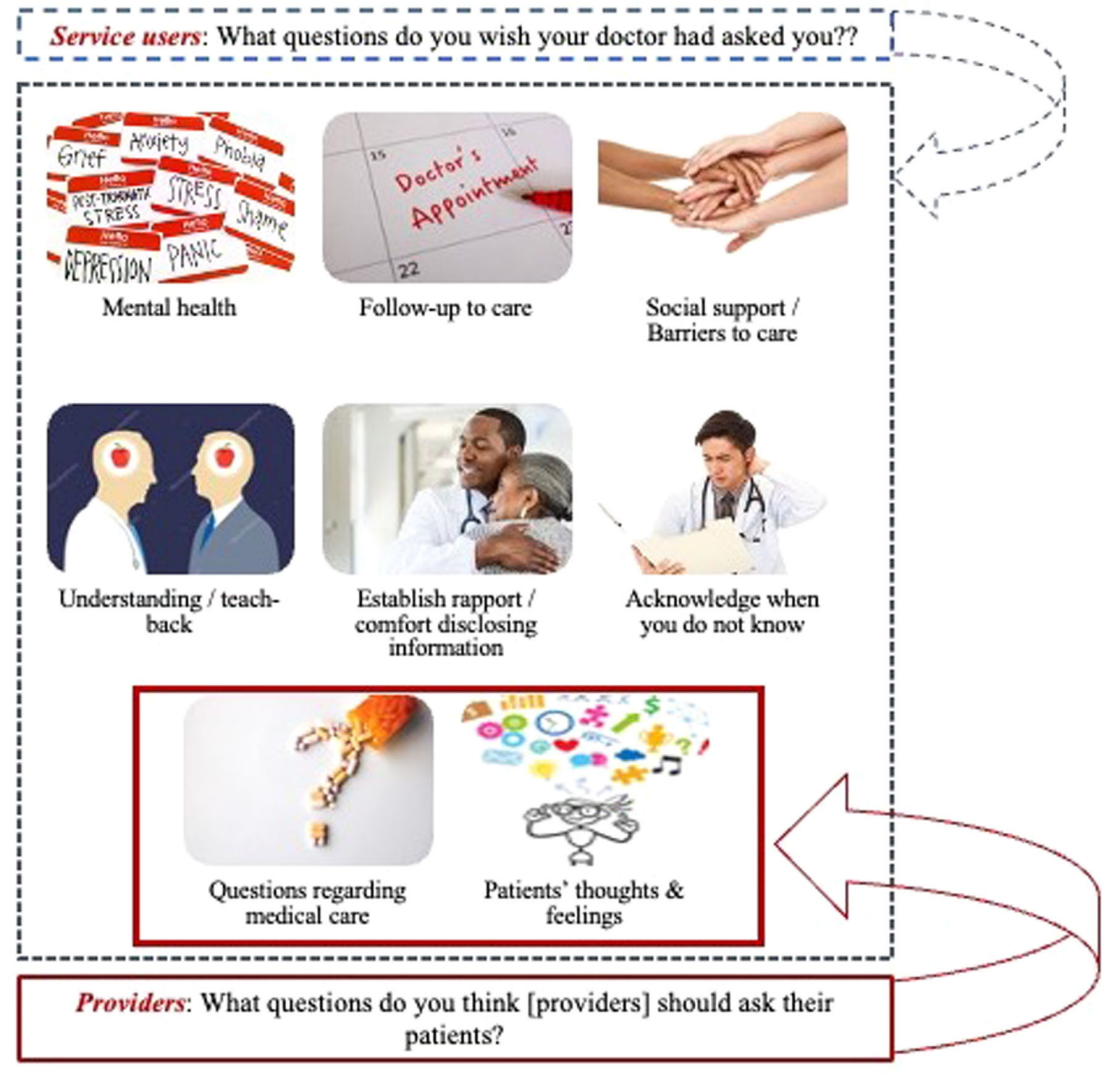
Healthcare experience—what questions do you wish your doctor had asked you? versus what questions do you think you should ask your patients?

Community residents and patients were asked, ‘As a patient, what questions do you wish your doctor had asked you?’ Several themes emerged in response to this question including mental health, medical care, follow‐up to care, social support and barriers to care, understanding of clinical diagnoses and terminology, patients' thoughts and feelings, questions that establish rapport and comfort to disclosing information and to acknowledge when providers are unsure about the issue discussed. Establishing rapport with patients and understanding their medical and nonmedical background and needs was one of the most prominent themes, though. On the contrary, when healthcare providers were asked, ‘From your perspective, what questions do you think [providers] should ask their patients?’ only two themes emerged: questions regarding medical care and questions on patients' thoughts and feelings. This discrepancy highlights the gap in what patients want or look for in their interactions with the provider and what providers actually think that relationship should look like. One explanation given by providers is that because of the short clinical encounter or interaction with the patient, they usually do not have enough time to go beyond the clinical care dimension (Figure 2).

## 4 DISCUSSION

This study investigated service users' and providers' viewpoints of the concept of health and compared the differences in expectations of service users and providers when discussing healthcare experiences. Several findings were uncovered and may have important implications for improvements in quality of care, patient–provider interaction and future research.

The discussion on the concept of health was met with participants expressing views that placed health as a top priority in their life. They also expressed a desire to live because they wanted to be around for their family and to enjoy life. We believe that this finding supports the importance of two‐way communication between providers and service users. Bidirectional communication would allow providers to gain an understanding of the level of importance that patients place on illness and their desire to avoid and overcome the effects that these illnesses could have on their ability to live.^5^ Additionally, this finding also builds on the qualitative findings of Naik et al.,^32^ in which we see the interconnectedness of self‐sufficiency, enjoyment, connection, balancing quality and length of life and engagement in care influence service users' desire to have health as their top priority.

Our research also highlighted the differences in users' expectations and what providers can offer. When discussing healthcare experiences, service users expressed a desire for providers to take the time to establish rapport and build trust. They want providers to view them as individuals, not just patients. These factors are instrumental in increasing the level of service users' satisfaction with their healthcare experiences.^33^ Additionally, it is evident from our study that building this relationship will present opportunities for service users and providers to discuss the social determinants of health. As seen by the focus group discussions, service users understand the impact that the social determinants of health, like housing and access to healthy food, can have on their health outcomes. This expectation that service users have for providers is often not met and results in providers having an altered perception of the stressors that patients face and low perceptions of the number of psychosocial problems that impact their health.^9^, ^13^ Therefore, it is our belief that meeting these patient expectations will result in better care coordination and adherence. If providers can gain greater insight into the plight of their patients, they will be able to connect them to the proper social services and assign treatment that is more feasible and better aligned with the patients' situation. This could also improve the quality of care (and life) by increasing medication adherence, decreasing the number of appointments missed, decreasing emergency department visits and decreasing hospital admissions and readmissions since the literature does demonstrate an association between life experiences and success in following medical advice.^34^, ^35^, ^36^, ^37^, ^38^, ^39^, ^40^, ^41^, ^42^ If patients are getting help on all of the social factors that cause them to place health at a lower priority, they will be able to achieve better health outcomes.^43^, ^44^

Lastly, our research demonstrated that physicians often focus on the good experiences that they have had with patients rather than the bad. Those good interactions are often defined by the provider assisting the service users with understanding their medication and their health condition. Providers expressed that these interactions allowed them to build relationships and empower patients to take control of their health. This finding supports the conclusion that providers perceive education as the key to gaining patient compliance with medication and increasing their trust of the medical system.^5^ Even though the providers' descriptions of healthcare experiences support previous research findings, it also emphasizes the disconnect between service users' and providers' expectations.

The findings from this study may be limited in its representation of different social identities (race, medical specialty, medical background, etc.) or individuals without health insurance coverage. Furthermore, this study might only be generalizable to other urban populations with similar demographics to Baltimore, Maryland. While this limitation impacts the ability to generalize to other settings or social identities, qualitative research is usually focused on in‐depth conversations and theme generation rather than generalizable findings. Despite this limitation, this study provides a unique perspective on service users' expectations versus the providers that they interact with. In particular, this study provides a unique strength in identifying the perspectives of both healthcare professionals and service users on healthcare factors that are most important for both groups and issues that are yet to be aligned.

## 5 CONCLUSION

When accessing healthcare quality, it is important for a health system to look beyond the effectiveness of their internal processes, the bad versus good outcomes of procedures and timeliness of care. Systems must take quality a step further and incorporate the expectations of service users when developing recommendations for quality improvement, beyond traditional patient satisfaction measures. We found that users' expectations and what providers can offer differ on a fundamental level. When asked questions about their healthcare experiences, service users primarily referred to their bad experiences whilst providers only mentioned the good experiences that they encountered with their patients. We also found that users desire conversations about the social determinants of health (i.e., housing, mental health, etc.) when visiting their provider. This emphasizes the need for providers to take the time to understand the social, cultural and economic factors that impact users' abilities to adhere to treatment plans. Improving the awareness of providers on these different factors can help to maximize the effectiveness of the healthcare system. Thus, the implications of this study are that there needs to be increased involvement of patients and service users in care administration and improvement in the dialogue between these parties. Additionally, this study also implies that there is a need for healthcare systems to further include additional personnel such as social workers and community health workers on their healthcare team to complement the care that providers are providing to their patients. By including this patient‐centred medical team in their healthcare system, organizations will be able to provide comprehensive care to patients and ensure that all of the patients' needs are being met.

## AUTHOR CONTRIBUTIONS

Nabil Natafgi contributed to the design of the study, data collection and analysis and writing of the manuscript. Olayinka Ladeji and Yoon Duk Hong contributed to data collection and analysis. Shanikque Blackwell contributed to data analysis and to writing of the manuscript. Gail Graham and Marcia Cort contributed to the design of the study and data collection. C. Daniel Mullins secured funding for the project and contributed to the design of the study. All authors reviewed and approved the final manuscript.

## CONFLICTS OF INTEREST

The authors declare no conflicts of interest.

## Supporting information

Supporting information.

## Data Availability

Data are available on request due to privacy/ethical considerations.
