# Supplementary material for: Similar values, different expectations: How do patients and providers view ‘health’ and perceive the healthcare experience?
Source: Health Expect. 2022 Apr 12;25(4):1517–28. doi: 10.1111/hex.13493 (PMC9327836; doi:10.1111/hex.13493)
Supplement: Supplementary file 1 — Supporting information. [file HEX-25--s001.docx]

# Supporting Information

Supporting Document 1: Interview Guide for Patients and Community Members

Supporting Document 2: Interview Guide for Health Care Professionals and Other Stakeholders

**Supporting Document 1: Interview Guide for Patients and Community Members**

Ice-Breaker

1. When learning new things, how do you like to learn? What tools do you use to make learning easy for you?
   1. [PROBE] Do you think a *community* (or an *organization*) can learn?
   2. [PROBE] If so, how? If not, why not?

The Concept of Health

1. Among the things that are of value to you, where do you place your health? Your family’s health?
   1. [PROBE] What motivates you to stay healthy?
   2. [PROBE] Do you reward yourself for doing things to be healthy? If so, how?
2. Do you know what your own health needs are?
   1. [PROBE] How do you learn how to stay healthy or manage your disease?
   2. [PROBE] Who or what provides that knowledge for you?
3. Do you want to live?
   1. [PROBE] Many people do not think about health until they do not have it. In other words, you may not value your health until you are diagnosed with a disease or become ill. How can we encourage people to focus on their health when they are healthy (i.e. not sick)?
   2. [PROBE] From your perspective, what can primary care providers and hospitals do differently to make people healthy? How can they support people to stay healthy?

Health Care Experience

1. I want you to go back, in your memory, to one great experience you, a family member, or a friend had with a doctor or a hospital. Now, briefly tell us what made it so great…
   1. [PROBE] Did that experience make you (or them) feel you (or they) had control of your (or their) healthcare? And what made you (or them) feel empowered?
2. As a patient, what questions do you wish your doctor had asked you?
   1. [PROBE] What is the most important question you feel your doctor hasn’t answered yet, to help you be healthy, stay healthy, or feel healthier?
   2. [PROBE] If you were a doctor, what question(s) would you ask a patient like you to be a better provider of care?

Concluding remarks

1. We thank you for your time, your voice and we are listening to you. In considering the goals we discussed earlier, is there anything we have not discussed that you want to tell us about? Anything we did not ask that you think we should consider?

**Supporting Document 2: Interview Guide for Health Care Professionals and Other Stakeholders**

Ice-Breaker

1. When learning new things, how do you like to learn? What tools do you use to make learning easy for you?
   1. [PROBE] Do you think a *community* (or an *organization*) can learn?
   2. [PROBE] If so, how? If not, why not?

The Concept of Health

1. Among the things that are of value to you, where do you place your health? Your family’s health?
   1. [PROBE] What motivates you to stay healthy?
   2. [PROBE] Do you reward yourself for doing things to be healthy? If so, how?
2. Do you know what your own health needs are?
   1. [PROBE] How do you learn how to stay healthy or manage your disease?
   2. [PROBE] Who or what provides that knowledge for you?
3. Do you want to live?
   1. [PROBE] Many people do not think about health until they do not have it. In other words, you may not value your health until you are diagnosed with a disease or become ill. How can we encourage people to focus on their health when they are healthy (i.e. not sick)?
   2. [PROBE] From your perspective, what can primary care providers and hospitals do differently to make people healthy? How can they support people to stay healthy?

Health Care Experience (10 minutes)

1. I want you to go back, in your memory, to one great experience you had with a patient. What did that experience look like?
   1. [PROBE] What did you specifically do to make the patient feel empowered?

1. From your perspective, what questions do you think doctors should ask their patients?
   1. [PROBE] ] What is one intervention that doctors would like to do to help their patients be healthy, stay healthy, or feel healthier?

Concluding remarks

1. We thank you for your time, your voice and we are listening to you. In considering the goals we discussed earlier, is there anything we have not discussed that you want to tell us about? Anything we did not ask that you think we should consider?
